# Artificial intelligence in the cath lab: Bridging predictive models and real-time procedural decision support

**DOI:** 10.17179/excli2026-9524

**Published:** 2026-08-06

**Authors:** Inderbir Padda, Shirobhi Sharma, Yashendra Sethi, Sneha Annie Sebastian, Harshan Atwal, Inderjeet Bharaj, Khushal Choudhary, Charles Sineri

**Affiliations:** 1 Department of Cardiology, One Brooklyn Health, Brooklyn, NY, USA; 2 Department of Cardiovascular Medicine, PearResearch, Dehradun 248001, India; 3 Department of Medicine, Subharti Medical College, Swami Vivekanand, Subharti University, Meerut, India; 4 Department of Internal Medicine, Augusta Health, Fisherville, West Virginia, USA; 5 Department of Internal Medicine, Saint James School of Medicine, Park Ridge, IL, USA; 6 Department of Internal Medicine, Abrazo Health Network, Glendale, AZ, USA; 7 Department of Interventional Cardiology, Icahn School of Medicine, Mount Sinai, New York, NY, USA; 8 Department of Cardiology, Richmond University Medical Center/Mount Sinai, Staten Island, NY, USA

**Keywords:** artificial intelligence, coronary computed tomography angiography, intravascular ultrasound, optical coherence tomography, percutaneous coronary intervention, risk prediction

## Abstract

The integration of Artificial Intelligence (AI) in medicine has been revolutionary, particularly in cardiology, where AI offers transformative tools for data integration, image analysis, and predictive modeling. In procedural settings such as percutaneous coronary intervention (PCI) planning, where timely decision-making is crucial, AI represents a promising avenue for real-time risk prediction. However, current clinical scores and risk models often fall short in dynamic environments like the catheterization (cath) lab due to their static nature and limited adaptability to intra-procedural complexities. Emerging AI models aim to leverage high-frequency physiological data, procedural metadata, and multimodal imaging to recognize evolving patterns and anticipate complications. Nevertheless, most existing applications remain retrospective, lack real-time integration, and are constrained by limited external validation. Looking ahead, the implementation of real-time AI systems in the cath lab holds significant potential to enhance procedural safety and outcomes by delivering anticipatory alerts and actionable insights that support clinical decision-making during PCI. However, it is important to note that most currently available AI models remain retrospective or observational in nature, and prospective evidence demonstrating improved clinical outcomes through real-time AI-guided interventions remains limited.

See also the graphical abstract(Fig. 1).

## Abbreviations

ACC: American College of Cardiology

ACS: acute coronary syndromes

AHA: American Heart Association

AI: artificial intelligence

AUC: area under the curve

BMC2: Blue Cross Blue Shield of Michigan Cardiovascular Consortium

CABG: coronary artery bypass graft

Cath: catheterization

CI-AKI: contrast-induced acute kidney injury

CHF: congestive heart failure

CTO: chronic total occlusions

CCTA: coronary computed tomography angiography

CNNs: convolutional neural networks

DL: deep learning

DRF: Distributed Random Forest

DNNs: Deep Neural Networks

FFR: fractional flow reserve

GBM: Gradient Boosting Machine

GRACE: Global Registry of Acute Coronary Events

IVUS: intravascular ultrasound

LAD: left anterior descending

LR: logistic regression

MACE: major adverse cardiac events

ML: machine learning

NCDR: National Cardiovascular Data Registry

OCT: optical coherence tomography

PCI: percutaneous coronary intervention

SVE: Soft Voting Ensemble Classifier

TIMI: Thrombolysis in Myocardial Infarction

XGBoost: Extreme Gradient Boosting

## Introduction

Percutaneous coronary intervention (PCI) success can be jeopardized by acute periprocedural complications, especially in high-risk lesion subsets. Accurate, timely risk prediction is essential to improving outcomes in the dynamic environment of the catheterization (cath) lab. Traditional scores such as the Global Registry of Acute Coronary Events (GRACE) and the Thrombolysis in Myocardial Infarction (TIMI) scores primarily rely on pre-procedural clinical variables to predict short-term mortality and major adverse cardiac events (MACE) (Rao et al., 2025[21]). While widely used, these models lack procedural granularity and fail to account for real-time intraprocedural changes. More advanced tools, such as the SYNTAX score and the National Cardiovascular Data Registry (NCDR) CathPCI, incorporate angiographic complexity and procedural urgency, thereby offering improved predictive performance (Palmerini et al., 2012[19]; Castro-Dominguez et al., 2021[4]).

However, these models remain static, are typically applied pre-procedurally, and are not responsive to evolving conditions during PCI (Palmerini et al., 2012[19]; Castro-Dominguez et al., 2021[4]). Moreover, existing risk scores are often generalized across diverse populations, limiting their precision for personalized, real-time decision-making. In contrast, artificial intelligence (AI) offers the capacity to analyze large, complex datasets and uncover nonlinear associations that traditional models may miss. By integrating multimodal imaging, hemodynamic parameters, operator inputs, and longitudinal patient data, AI models hold promise for delivering real-time risk forecasting during PCI.

Nevertheless, most published models have been developed and validated using retrospective datasets and should currently be viewed as hypothesis-generating tools rather than established real-time clinical decision-support systems. However, several barriers remain to clinical implementation, including data standardization, model interpretability, and seamless workflow integration. Additional challenges include interoperability between imaging and hemodynamic platforms, external validation across diverse healthcare systems, regulatory approval pathways, model drift over time, cybersecurity considerations, and clinician acceptance, all of which must be addressed before widespread clinical deployment becomes feasible.

Several recent reviews have examined the broader applications of AI in PCI and interventional cardiology. However, the present review specifically focuses on the emerging concept of real-time AI-enabled procedural risk prediction within the catheterization laboratory. Particular emphasis is placed on distinguishing retrospective predictive models from true intraprocedural decision-support systems, evaluating the feasibility of workflow integration, and highlighting the technical, operational, and regulatory challenges that must be overcome before real-time AI can be implemented in routine clinical practice. This review explores the evolving landscape of AI-based risk prediction in the cath lab, assesses current models and limitations, and outlines future directions for achieving real-time, actionable forecasting of procedural complications.

## Methodology

This review was conducted as a narrative synthesis based on targeted searches of PubMed/MEDLINE and Embase, supplemented by hand-searching reference lists from landmark PCI risk models, AI and machine learning-based PCI studies, and relevant guideline and consensus documents. Search terms encompassed PCI, procedural complications, catheterization laboratory workflow, and AI/ML concepts, including “real-time,” “intra-procedural,” “hemodynamics,” “IVUS,” and “OCT.” Priority was given to higher-quality and clinically applicable evidence, including prospective registries, multicenter datasets, and externally validated models when available. Findings were synthesized qualitatively, with emphasis on feasibility for real-time deployment and integration into cath-lab workflows.

## Current Landscape of Risk Prediction in Interventional Cardiology

Despite the growing promise of machine learning (ML) models in interventional cardiology, the foundation of risk assessment in interventional practice still relies heavily on established clinical risk scores. These tools, endorsed in major guidelines, remain integral to therapeutic decision-making in acute coronary syndromes (ACS) and PCI. However, traditional risk scores offer only a static profile of patient risk. Recognizing their strengths and limitations is key to identifying opportunities for more adaptive, real-time approaches to procedural decision-making.

### Traditional risk scores

Current guidelines from the American College of Cardiology (ACC) and the American Heart Association (AHA) endorse the GRACE and TIMI scores for risk stratification of short-term mortality, MACE, and for guiding therapeutic decision-making in acute coronary syndromes (Rao et al., 2025[21]). Among these, the GRACE score has demonstrated superior predictive accuracy for mortality and myocardial infarction, with a c-statistic of 0.812 (95 % confidence interval [CI], 0.772-0.851), compared to the more subjective physician-estimated risk, which has a c-statistic of 0.652 (95 % CI, 0.596-0.708), highlighting the value of objective risk assessment tools (Chew et al., 2013[5]).

However, traditional risk scores like GRACE and TIMI focus predominantly on clinical and laboratory variables, omitting angiographic and procedural characteristics that may enhance individualized risk prediction (Palmerini et al., 2012[19]). To address this limitation, alternative models such as the SYNTAX score and the NCDR CathPCI score incorporate anatomical complexity and procedural urgency (Palmerini et al., 2012[19]; Castro-Dominguez et al., 2021[4]). The SYNTAX score evaluates factors such as lesion location, number of diseased vessels, chronic total occlusion (CTO), and involvement of the proximal Left anterior descending (LAD) or left main artery (LMA) (Palmerini et al., 2012[19]). Meanwhile, the NCDR CathPCI risk score integrates both angiographic and procedural variables, including the presence of cardiogenic shock and emergent procedural context, to improve perioperative risk assessment and improve patient selection for procedures (Castro-Dominguez et al., 2021[4]).

Notably, the NCDR CathPCI model has demonstrated superior predictive performance for in-hospital mortality, with a c-statistic of 0.89 (95 % CI, 0.87-0.92), compared to the GRACE score's 0.79 (95 % CI, 0.75-0.83), despite GRACE being a recommended tool in current ACC/AHA guidelines (Parco et al., 2021[20]). These added dimensions enable a more granular assessment of procedural complexity and patient-specific risk, which may better support decision-making during PCI.

Although these c-statistics demonstrate strong discrimination within their respective derivation and validation cohorts, they were generated in specific clinical contexts, including predominantly ACS populations, pre-procedural risk assessment, and registry-based environments. As such, their performance may not generalize to intraprocedural decision-making, contemporary cath-lab workflows, or diverse patient populations with varying lesion complexity, operator strategies, and institutional practices.

### Model paradigms: Predictive vs. Real-time interpretive AI

In interventional cardiology, AI models can be broadly categorized into predictive models and real-time interpretive models. Predictive models are typically trained on retrospective datasets and used to estimate the likelihood of future outcomes such as mortality, MI, or MACE. These models operate at defined timepoints, primarily in the pre-procedural phase, and commonly employ algorithms such as logistic regression, random forest, or gradient boosting.

Importantly, the majority of AI studies currently reported in PCI fall into the predictive category. Although these models frequently demonstrate strong discrimination metrics, such as high AUC or c-statistic values, they are generally derived from retrospective datasets and evaluated offline. Consequently, strong predictive performance does not necessarily indicate feasibility, reliability, or clinical benefit in a live intraprocedural environment.

By contrast, real-time interpretive models are designed to process continuous intra-procedural data streams, including hemodynamic waveforms, intravascular imaging inputs such as IVUS and OCT, and device-related metrics. These models require low-latency computing infrastructure and are optimized for high-frequency data processing. Techniques such as convolutional neural networks (CNNs) for image interpretation and recurrent neural networks for sequential physiologic data enable real-time inference.

This distinction is critical for clinical integration. Predictive models support pre-procedural planning by estimating overall patient risk, while real-time models provide proceduralists with dynamic insights as the intervention unfolds, facilitating adaptive and time-sensitive decision-making within the cath lab.

### AI models

ML models are a branch of AI that enables computers to learn patterns from data and continually adjust and enhance their performance as they are provided with an increasing amount of data (Jiang et al., 2020[15]). Current ML approaches being studied include the Gradient Boosting Machine (GBM), Distributed Random Forest (DRF), Logistic Regression (LR), Soft Voting Ensemble Classifier (SVE), and Deep Learning (DL) (Tofighi et al., 2024[23]; Sherazi et al., 2021[22]). DL models are built on Deep Neural Networks (DNNs); these artificial neural networks can perform hierarchical feature learning, which enables the integration of ECG patterns, imaging analysis, and patient vitals to predict post-PCI events (Jha and Topol, 2016[14]). These models are being studied for a range of applications, including personalized procedural risk prediction, outcome forecasting, automated analysis of angiographic and intravascular imaging, and potential intraprocedural decision support (Jha and Topol, 2016[14]; Khalpey et al., 2025[16]).

There is a substantial amount of datasets being provided to ML models; however, the most common include the National Cardiovascular Data Registry (NCDR) CathPCI Registry, the Blue Cross Blue Shield of Michigan Cardiovascular Consortium (BMC2) registry, the Mayo Clinic PCI registry, and the National Inpatient Sample (NIS) (Hamilton et al., 2024[12]; Galimzhanov et al., 2023[11]; Zaka et al., 2024[27]). These registries provide ML models with large sample sizes and extensive variables, including demographics, comorbidities, procedural details, laboratory values, and imaging findings, as well as robust outcome ascertainment, enabling the development and validation of predictive models for a range of post-PCI outcomes (Hamilton et al., 2024[12]; Galimzhanov et al., 2023[11]; Zaka et al., 2024[27]).

### Retrospective AI models

In a study aimed at developing a prediction model for 30-day mortality following PCI, an ML model integrating the Veterans Affairs (VA) SYNTAX score along with presentation status (salvage, emergent, urgent), ST segment elevation myocardial infarction (STEMI), cardiogenic shock, age, congestive heart failure (CHF), prior valve disease, chronic kidney disease (CKD), chronic lung disease, atrial fibrillation, international normalized ratio (INR), body mass index (BMI), hemoglobin level, and previous coronary artery bypass graft (CABG) surgery outperformed the traditional NCDR CathPCI (Doll et al., 2021[8]). The 14 variables that comprise the final model were filtered and selected by six separate ML models using regression strategies and relative influence scores from 50 original covariates generated by the Veterans Affairs Clinical Assessment Reporting and Tracking (CART) application (Doll et al., 2021[8]). From the 70,503 PCIs performed in VA hospitals, the CART PCI mortality model had superior predictability with a c-index of 0.92 (95 % CI, 0.91-0.94) compared to the 0.85 (95 % CI, 0.83-0.86) of NCDR CathPCI in 30-day mortality (Doll et al., 2021[8]). Furthermore, the CART PCI model had a positive likelihood ratio of 10.86 for predicting mortality events in patients in the top decile of risk, compared to 7.99 for the NCDR model, emphasizing the significant improvement in predictability post-PCI over traditional risk scores (Doll et al., 2021[8]).

The no-reflow phenomenon (NRF) is a significant post-procedural complication in patients undergoing PCI. Therefore, accurate risk prediction of NRF will help improve pre-procedural planning and clinical decision-making. In a study involving 261 patients, an LR-XGB model was used to predict no-reflow phenomena in patients post-PCI, with an AUC of 0.829 (95 % CI, 0.779-0.880) (Wang et al., 2024[25]). The model incorporated stent diameter, serum potassium, Killip class II-IV, thrombotic aspiration, prophylactic tirofiban, troponin I, and thrombolysis in myocardial infarction flow after initial balloon dilation (TIMI flow after initial balloon dilation, TFAID) (Wang et al., 2024[25]).

In a study using a random forest algorithm, Deng et al. constructed a model to predict the no-reflow process and in-hospital mortality. The model demonstrated optimal discrimination for the NR phenomenon, with an AUC of 0.7891 (95 % CI: 0.7093-0.8688), and an AUC for in-hospital mortality of 0.9273 (95 % CI: 0.8819-0.9728) (Deng et al., 2022[7]). Another study by Fedai et al. incorporated the CALLY index into an Extreme Gradient Boosting (XGBoost) ML model; the model achieved a no-reflow detection rate of 78.11 %, demonstrating the value of the CALLY index as a predictive biomarker when combined with ML techniques (Fedai et al., 2024[9]). In a study on over 84,000 patients that underwent coronary procedures, an XGBoost ML model was trained to identify predictors of catheter-induced coronary and aortic dissection. This low-incidence complication can adversely affect inpatient mortality (Klaudel et al., 2022[17]). The model demonstrated an F1 score of 0.488, indicating a balance between precision and recall in identifying high-risk patients (Klaudel et al., 2022[17]). The key predictors in this model included use of a guiding catheter, small or stenotic ostium, radial access, hypertension, acute myocardial infarction, prior angioplasty, female sex, chronic renal failure, atypical coronary origin, and chronic obstructive pulmonary disease (Klaudel et al., 2022[17]).

A study conducted by Wenzl et al. aimed to improve the GRACE 2.0 score by incorporating sex differences in disease characteristics. The GRACE 3.0 used an XGBoost ML model to achieve an AUC of 0.91 (95 % CI, 0.89-0.92) in the external cohort validation for in-hospital death among male patients and 0.87 (95 % CI, 0.84-0.89) among female patients. These results indicate improved discrimination with the GRACE 3.0 score, particularly in women, compared with the original GRACE 2.0 score, with AUCs of 0.86 (95 % CI 0.86-0.86) in male patients and 0.82 (95 % CI 0.81-0.82) in female patients. The ML-based GRACE 3.0 score offers improved predictive performance in both sexes, accounting for sex differences in risk stratification. More importantly, it holds the potential to address structural inequities in the management of female patients with NSTE-ACS, elucidating a more equitable and effective approach to patient care (Wenzl et al., 2022[26]).

### Prospective AI models

In a prospective PCI registry study involving 2,242 patients, a CatBoost-based ML model, composed of 140 clinical parameters, outperformed risk scores, including GRACE, ACEF, SYNTAX II, and TIMI scores, in predicting 3-year all-cause mortality and 3-year cardiovascular cause mortality (Călburean et al., 2022[3]). The ML model superiorly predicted 3-year all-cause mortality with an area under the curve (AUC) of 0.854 (95 % CI, 0.835-0.875), compared to the AUCs of 0.762, 0.764, 0.730, and 0.691 for GRACE, ACEF, SYNTAX II, and TIMI, respectively (Călburean et al., 2022[3]). The AUC for 3-year cardiovascular mortality prediction was 0.886 (95 % CI, 0.867-0.901) for the ML model, compared to AUCs of 0.797, 0.792, 0.757, and 0.696 for GRACE, ACEF, SYNTAX II, and TIMI, respectively (Călburean et al., 2022[3]). The significant increase in predictive value highlights the potential of ML models to enhance post-PCI patient care and management, as they can analyze and compute a broad range of variables that traditional risk models currently do not.

## Need for Real-Time AI Integration in the Cath Lab

While existing studies demonstrate high predictive accuracy using retrospective or pre-procedural datasets, these models remain limited to static predictions and have yet to achieve the real-time forecasting capability required for intra-procedural decision support. The integration of AI into dynamic, real-time decision-making during PCI, therefore, remains an evolving frontier. Interventionalists are often faced with a dynamic and high-stakes environment during PCI, influenced by the severity of the lesion and complexity of the case, where clinical status, procedural strategy, and physiological parameters can change by the minute. During PCI, data streams continuously from hemodynamic monitors, angiographic systems, intravascular imaging, and interventional device platforms. This information includes blood pressure, heart rate, contrast volume, stent deployment metrics, and real-time imaging outputs such as IVUS and OCT. The complexity arising from the simultaneous interplay of these variables makes periprocedural risk difficult to predict using pre-procedural models alone.

**Figure 2:**
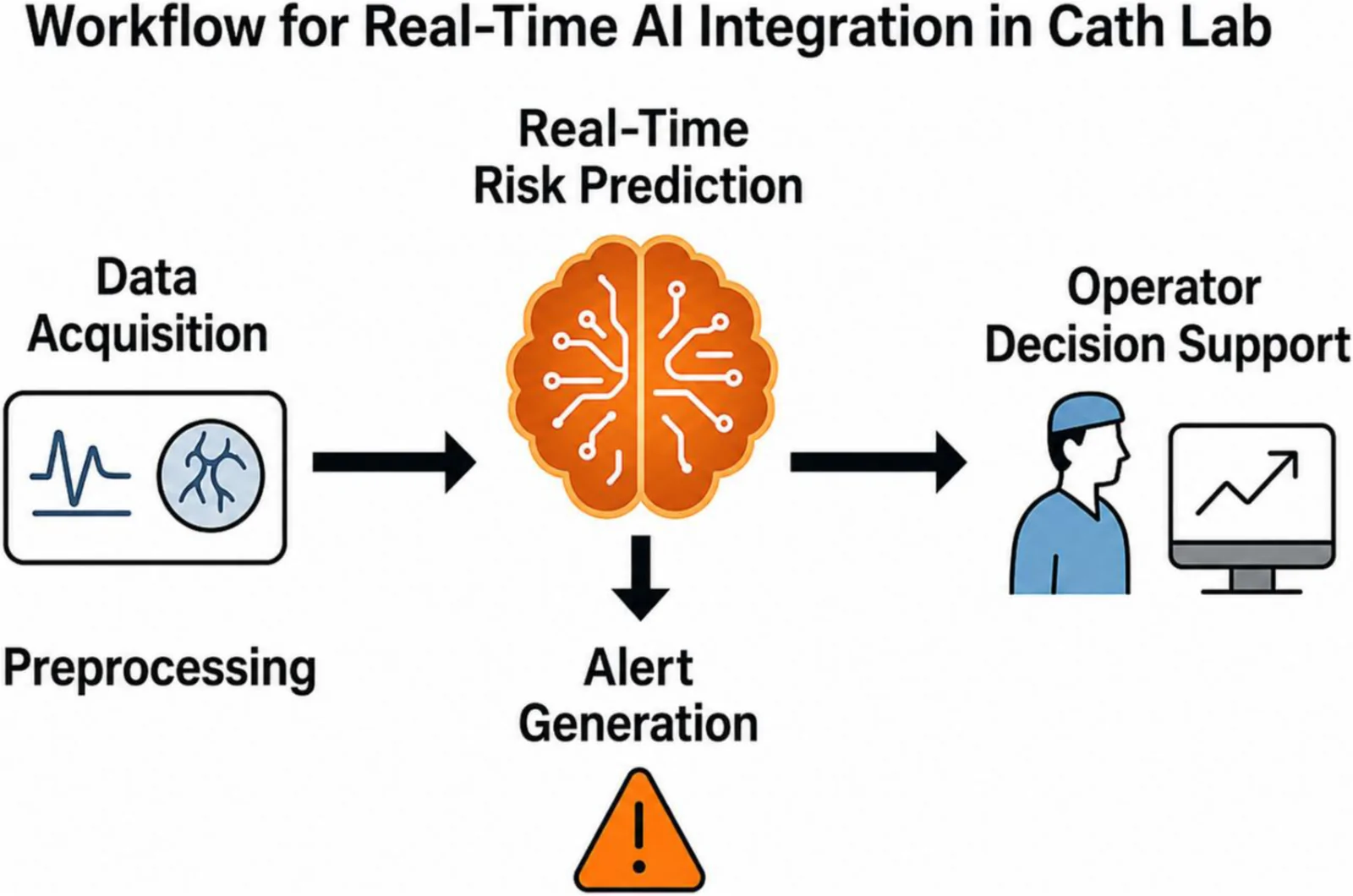
This figure depicts diverse data acquisition to support dynamic risk prediction and clinical decision-making during percutaneous coronary intervention (PCI).

Traditional risk scores are typically applied prior to the procedure and lack the ability for intraprocedural updates. These models rely heavily on pre-procedural clinical and anatomical factors and fail to incorporate evolving intraprocedural data, such as device manipulations or contrast-induced changes in renal function or vascular integrity. There is a need for real-time AI-driven risk prediction tools that are capable of interpreting data as procedures are underway and providing the proceduralist with changes in patient risk. However, such systems remain largely conceptual or in early developmental stages, and few have been prospectively evaluated within routine clinical workflows. Such models would need to continuously aggregate high-frequency data streams and apply ML algorithms (Figure 2(Fig. 2)).

Current early development AI models have shown promise and have often outperformed traditional risk scores in retrospective analyses and selected prospective observational studies. However, evidence demonstrating that these models can safely and effectively influence real-time procedural decision-making remains limited. Contemporary literature reports that prospective models demonstrate superior predictive capabilities when trained on bigger datasets with hundreds of clinical and procedural variables. Such models include those using CatBoost and deep neural networks. However, the implementation of AI in real-time decision support systems remains limited due to challenges in data standardization, processing latency, and the lack of integration with existing cath lab infrastructure.

## Existing or Emerging Real-Time AI Approaches

### Machine learning models with dynamic input

AI models can automate and enhance real-time image analysis, lesion assessment, and procedural guidance by integrating data from physiologic monitors (e.g., pressure wires, flow sensors), contrast injectors, and advanced imaging modalities. In the context of IVUS and OCT, ML models facilitate automated segmentation, plaque morphology analysis, and stent optimization, providing rapid, reproducible, and quantitative assessments that can be integrated with angiographic data for comprehensive procedural guidance (Avram et al., 2023[2]). These findings can be co-registered with angiographic images to guide wire crossing and stent placement, potentially reducing the use of contrast and radiation exposure. In physiologic monitoring, ML models have been developed that can analyze pressure and flow data to automate the assessment of lesion significance, potentially reducing operator variability and expediting decision-making (Veneziano et al., 2025[24]). For contrast injectors, ML can optimize contrast dosing and timing, aiming to minimize nephrotoxicity and reduce overall contrast use, as highlighted by the American Heart Association (Armoundas et al., 2024[1]).

### Image-based AI models

Real-time angiogram analysis during PCI is increasingly supported by computer vision and CNNs, which enable automated detection of flow reduction, vessel segmentation, and complications such as dissections (Iyer et al., 2021[13]). DL architectures have demonstrated high accuracy and rapid processing for segmenting coronary arteries, quantifying stenosis, and identifying lesion morphologies such as dissection and thrombosis, within seconds (Iyer et al., 2021[13]). Although these technical capabilities suggest potential suitability for intraprocedural support, most studies have evaluated performance retrospectively or in controlled development settings rather than in fully integrated live clinical workflows. Modern neural network models, such as those based on Faster-RCNN and ResNet architectures, can achieve high F1 scores (up to 0.96) and prediction rates suitable for real-time applications, feasible for intra-procedural support (Danilov et al., 2021[6]). These systems can automatically generate diagnostic maps, flag lesion severity, and provide quantitative, reproducible assessments that reduce inter-operator variability. Additionally, integrating these AI tools with dynamic road mapping and catheter tracking can enhance visual guidance and potentially reduce the need for contrast use during interventions (Ma et al., 2020[18]).

### Real-time decision support tools

Early prototypes and commercially piloted tools have demonstrated that with careful integration, AI can provide real-time risk assessments and procedural guidance without disrupting workflow. A retrospective study by Fischer et al. involving 642 patients demonstrated that integrating ePRISM, an AI-driven clinical decision support tool, into the PCI workflow significantly reduced periprocedural complications. Fully embedded within the electronic health record, ePRISM automatically extracted preprocedural patient data to generate individualized risk assessments and contrast volume recommendations. Nursing staff reviewed these assessments preoperatively and coordinated hydration protocols accordingly. On the day of the procedure, risk scores and contrast thresholds were communicated during the procedural time-out. Intraoperatively, ePRISM provided alerts when contrast use approached 80 % of the recommended limit, enabling dynamic fluid management. Postprocedural hydration was tailored based on total contrast volume and hemodynamic status. The implementation of ePRISM was associated with a significant reduction in contrast-induced acute kidney injury (CI-AKI), from 10 % to 2.18 % (P < .0001), along with fewer bleeding complications and a decrease in the average hospital length of stay from 3.44 to 1.79 days. While ePRISM represents an important example of AI-enabled clinical decision support, it should be noted that the system primarily utilizes preprocedural and peri-procedural risk assessment workflows rather than continuous intraprocedural ML-based adaptation. Consequently, it highlights the feasibility of workflow-integrated AI but does not fully represent autonomous real-time procedural risk forecasting. These findings support the feasibility and clinical value of real-time AI integration in improving safety and outcomes during PCI without disrupting procedural workflow (Fischer et al., 2025[10]).

## Technical and Operational Challenges

Although the potential of AI in the cath lab is considerable, significant technical and operational challenges hinder its ability to deliver real-time risk prediction during procedures. Accurate risk forecasting in dynamic PCI environments requires continuous, high-quality data from hemodynamic monitors, intravascular imaging, and procedural devices. The majority of cath labs operate independently with already established formats, which can fragment datasets and cause discrepancies that may degrade model performance for predictive modeling. Standardization of data acquisition from different platforms is essential in achieving real-time utility in the cath lab.

Real-time risk prediction depends on the ability to continuously aggregate and process incoming data during a procedure. In order to achieve this, the integration of AI models in the cath lab must be swift and be embedded smoothly into the current cath infrastructure. The models should be developed to work seamlessly with interventional imaging systems, physiological monitors, and EMR. Addressing this will require a collaborative approach involving device manufacturers, software engineers and developers, and healthcare institutions. The goal is to develop interoperable systems that allow AI tools to interpret evolving clinical data and deliver timely, actionable risk assessments to guide decision-making without disrupting procedural workflows.

The catheterization lab environment demands extremely efficient data processing for AI tools to be clinically useful. AI models, particularly those based on deep neural networks, are computationally intensive when processing high-frequency, multimodal inputs such as live imaging and physiological waveforms. To enable real-time risk prediction, these models must operate on low-latency computing infrastructure supported by optimized hardware accelerators. The system must be efficient enough to sustain real-time performance without compromising predictive accuracy.

Additional barriers to implementation extend beyond technical integration. Regulatory approval frameworks for continuously learning AI systems remain under development, and requirements for transparency, explainability, and post-market surveillance may affect deployment timelines. Furthermore, model performance may deteriorate over time due to dataset shift and evolving clinical practice patterns, requiring continuous monitoring and recalibration. Operator trust, usability, and medicolegal accountability also represent critical determinants of successful adoption. These challenges suggest that widespread implementation of real-time AI in routine PCI is unlikely to occur without substantial multidisciplinary collaboration and prospective validation.

## Future Directions and Research Priorities

Future perspectives in AI-enhanced interventional cardiology focus on transforming retrospective models into operational real-time systems capable of supporting procedural decision-making. At present, however, evidence supporting AI-driven modification of procedural strategy remains limited, and future studies should prioritize demonstrating clinical utility rather than predictive performance alone.

Standardizing data formats across various platforms, such as real-time hemodynamic parameters, intraprocedural imaging, device feedback, and EMR into a centralized platform, would enable the processing of temporally aligned high-frequency inputs during PCI. Real-time, actionable AI recommendations in the cath lab are essential and will require the development of closed-loop models capable of modifying procedural strategies in response to detecting changes in anatomical and physiological parameters.

While contemporary AI models have shown promise, most are validated retrospectively, limiting their applicability in real-world settings. This further stresses the need for large, multicenter prospective studies to evaluate performance, reliability, and clinical impact across diverse populations. Randomized and prospective implementation studies will be necessary to determine whether AI-assisted workflows improve patient outcomes, procedural efficiency, or operator decision-making compared with contemporary standard care.

For AI to be implemented successfully in real time, it must be embedded within existing cath lab software without introducing workflow disruptions. Models should be optimized for low-latency processing, intuitive real-time visualization, and user-friendly interfaces that support the proceduralist's day-to-day clinical activities.

## Limitations

Most current AI models in interventional cardiology are trained on retrospective datasets, limiting their utility for real-time clinical application. Furthermore, many studies cited as supporting future real-time AI applications are based on pre-procedural or retrospective analyses rather than live intraprocedural systems. As a result, the distinction between predictive modeling and genuine real-time clinical decision-support remains blurred in the current literature, and strong predictive performance in retrospective studies may not necessarily translate into successful real-world implementation.

External validation across diverse populations remains limited, raising concerns about generalizability, while incomplete integration into cath lab workflows and poor model interpretability continue to hinder adoption. As this is a narrative review without a predefined protocol or formal bias assessment, selection bias may be present; however, efforts were made to include diverse study designs and prioritize externally validated, clinically relevant models.

Beyond technical barriers, the implementation of real-time AI in the catheterization laboratory introduces regulatory, legal, and data governance challenges. Adaptive algorithms that evolve post-deployment may face stricter oversight, and medicolegal responsibility for AI-guided decisions remains ill-defined, highlighting the need for clinician-supervised frameworks. Continuous access to sensitive physiologic and imaging data also necessitates robust cybersecurity, data governance, and audit mechanisms. Addressing these ethical and operational issues alongside technical validation is essential for safe and responsible clinical deployment.

## Conclusions

There remains a critical unmet need for real-time procedural risk forecasting and decision support during PCI, as traditional scores are limited by their static nature and lack of intra-procedural adaptability. AI offers a compelling framework for enabling dynamic analysis of multimodal data that may ultimately support complication prediction and procedural decision-making in real time. However, current evidence remains largely retrospective, and few systems have demonstrated clinical benefit within prospective interventional studies. While early AI models demonstrate promise, their clinical utility is constrained by retrospective design, limited external validation, and integration challenges. Moving forward, success will depend on rigorous prospective testing, seamless interoperability with cath lab systems, and clinician-centered design to ensure feasibility. If validated through rigorous prospective studies and successfully integrated into clinical workflows, real-time AI may contribute substantially to improving procedural safety and precision in interventional cardiology. However, its role in routine PCI practice remains investigational at present.

## Declaration

### Conflict of interest

The authors declare that they have no disclosures or conflict of interest. Also, the authors declare that we have no known competing financial interests or personal relationships that could have appeared to influence the work reported in this paper.

### Acknowledgments

None.

### Funding

This research did not receive any specific grant from funding agencies in the public, commercial, or not-for-profit sectors.

### Artificial Intelligence (AI) - assisted technology

Artificial intelligence was not used in the preparation of this manuscript.

### Ethics approval

Not applicable.

### Author contributions

**IP, YS, SAS:** Conceptualization, writing - original draft, writing - review and editing; **SS:** Writing - original draft, writing - review and editing; **HA:** Writing - original draft, writing - review and editing; **IB:** Review, editing, and submission process; **KC:** Writing - original draft, writing - review and editing; **CS:** Supervision, review, editing, and final approval of manuscript

## Figures and Tables

**Figure 1:**
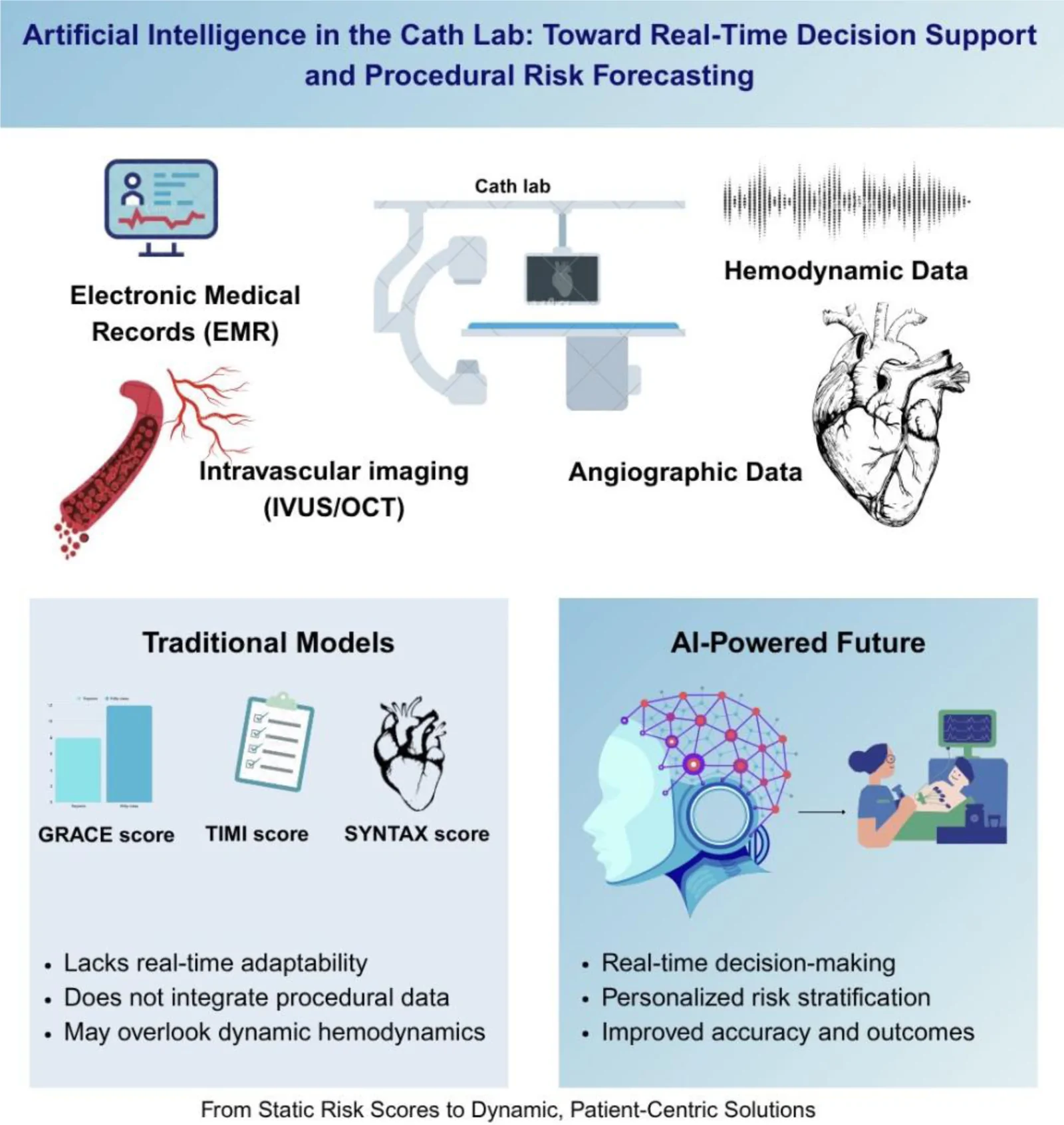
Graphical abstract
